# Genotoxic and Cytotoxic Effects of Antiretroviral Combinations in Mice Bone Marrow

**DOI:** 10.1371/journal.pone.0165706

**Published:** 2016-11-02

**Authors:** Aroldo Vieira de Moraes Filho, Cláudia de Jesus Silva Carvalho, Cristiene Costa Carneiro, Camila Regina do Vale, Débora Cristina da Silva Lima, Wanessa Fernandes Carvalho, Thiago Bernardi Vieira, Daniela de Melo e Silva, Kênya Silva Cunha, Lee Chen-Chen

**Affiliations:** 1 Laboratório de Radiobiologia e Mutagênese, Departamento de Genética, Instituto de Ciências Biológicas (ICB), Campus Samambaia, Universidade Federal de Goiás (UFG), Caixa Postal 131, 74001–970, Goiânia, GO, Brazil; 2 Programa de Pós-Graduação em Ecologia e Conservação, Universidade do Estado de Mato Grosso, Campus Universitário de Nova Xavantina, BR 158, Caixa Postal 8, 78.690–000, Nova Xavantina, MT, Brazil; University of California, San Francisco, UNITED STATES

## Abstract

Commonly used guidelines for the management of human immunodeficiency virus (HIV) infection (highly active antiretroviral therapy, HAART) include drug combinations such as tenofovir disoproxil fumarate (TDF) + lamivudine (3TC) and combivir [zidovudine (AZT) + 3TC] + efavirenz (EFV). These combinations may enhance the genotoxic effects induced by such drugs individually, since the therapy requires lifelong adherence and the drugs have unknown effects during treatment. Thus, the evaluation of the benefits and risks of HAART is of great importance. In order to assess the cytotoxic and genotoxic potential of three concentrations of each of the antiretroviral combinations TDF + 3TC (800 + 400, 1600 + 800, and 3200 + 1600 mg/kg body weight, BW) and combivir + EFV (200 + 100 + 400, 400 + 200 + 800, and 800 + 400 + 1600 mg/kg BW) after two exposure periods (24 h and 48 h), in the present study the *in vivo* comet assay (single-cell gel electrophoresis) and the mouse bone marrow micronucleus test were used. Neither TDF + 3TC nor combivir + EFV induced DNA damage at any concentrations tested after 24 h or 48 h using the comet assay. After 24 h, both combinations increased the micronucleus frequency at all concentrations tested. After 48 h, combivir + EFV increased the micronucleated polychromatic erythrocyte (MNPCE) frequency at the two highest concentrations tested. Polychromatic erythrocytes (PCE)/normochromatic erythrocytes (NCE) ratio was high for both combinations, suggesting that they can be mitogenic. Since genotoxicity may be related to carcinogenesis, it is necessary to conduct further studies to verify the long-term mutagenic effects of these drugs.

## Introduction

The fact that the human immunodeficiency virus (HIV) undergoes several mutations in DNA structure hinders the success of treatments with only one drug. Additionally, in recent years, little progress has been made towards the development of an HIV vaccine, and the maximum efficiency achieved was 31.2%. Thus, the highly active antiretroviral therapy (HAART), a combination of two or more antiretroviral drugs, has been used effectively and safely in the management of HIV/AIDS since 1996 [1–4].

Common guidelines for the management of HIV infection (HAART) include the following drug combinations: tenofovir disoproxil fumarate (TDF) plus lamivudine (3TC) and efavirenz (EFV) plus combivir [zidovudine (AZT) + 3TC] [5–8]. However, in studies of each of these drugs individually, some of them presented several side effects. TDF and EFV caused hepatocellular adenomas, carcinomas, and pulmonary alveolar/bronchiolar adenomas in female mice. AZT had clastogenic effects such as sister chromatid exchange and reduction in telomere length. Finally, 3TC exhibited clastogenic effects with micronuclei induction [9–16].

Consequently, these combinations may enhance the genotoxic effects induced by the drugs individually, due to the requirement of lifelong adherence and the unknown effects of long-term treatment. Moreover, the evaluation of the risk/benefit of the drugs should always be performed, because even though many antiretroviral combinations apparently do not present risk to human health due to low levels of toxicity, the cumulative effects of the treatment over decades is still controversial and not fully understood [4,17–20]. Considering that genotoxicity may be related to carcinogenesis, it is important to evaluate the genotoxic effects of medicines using tests such as the comet assay and the micronucleus test.

The comet assay is useful for detecting DNA damage caused by alkylating, intercalating, and oxidizing agents. The alkaline version of the test detects DNA single- and double-strand breaks, alkali-labile sites, and crosslinks, lesions that can be repaired, since they have not gone through repair mechanisms [21–25].

The micronucleus test detects DNA damage caused by clastogenic and aneugenic agents by assessing DNA damage at the chromosome level. Micronuclei represent the genetic material lost by the main core due to the action of physical, chemical, or biological agents that caused genetic damage to the chromosome [26–29].

Therefore, the aim of the present study was to assess the cytotoxic and genotoxic potential of the antiretroviral combinations combivir + EFV and TDF + 3TC using the comet assay and the mouse bone marrow micronucleus test [26,30,31].

## Materials and Methods

### Animals

This study was approved by the Animal Research Ethics Committee of the Universidade Federal de Goiás (CEUA/UFG no. 046/13) and followed the rules of animal management and experimentation of the Colégio Brasileiro de Experimentação Animal [32]. Healthy, young adult outbred male mice (*Mus musculus*, Swiss Webster), weighing 30–40 g, aged 7–12 weeks, obtained from the Central Laboratory of the Universidade Federal de Goiás were brought to the laboratory 7 days prior to the experiment. They were housed in polypropylene cages (40 cm × 30 cm × 16 cm), lined with wood shavings, changed daily, with five animals each, at 24 ± 2°C, 50 ± 20% humidity, and a light-dark natural cycle of 12 h. The animals were fed with standard food pellets (appropriate commercial rodent diet Labina, Ecibra Ltda, Santo Amaro, SP, Brazil) and water was provided *ad libitum*.

### Chemicals

The medicines Viread^®^ [300 mg TDF (CAS 202138-50-9) per tablet], Lamivudina^®^ [150 mg 3TC (CAS 134678-17-4) per tablet], Estiva-600^®^ [600 mg EFV (CAS 154598-52-4) per tablet], and Combivir^®^ [300 mg AZT (CAS 30516-87-1) + 150 mg 3TC per tablet], kindly donated by the Hospital de Doenças Tropicais Dr. Anuar Auad, Goiânia, GO, Brazil, were used in this study. Fetal calf serum (Laborclin, Pinhais, PR, Brazil), Giemsa (Newprov, Pinhais, PR, Brazil), dibasic sodium phosphate, monobasic sodium phosphate, absolute methanol, NaCl (Vetec Química Fina Ltda, Duque de Caxias, RJ, Brazil), agarose normal melting point, agarose low melting point, phosphate buffered saline (PBS), triton X-100, dimethyl sulfoxide (DMSO), stock lysis solution, tris-HCl buffer, ethidium bromide (Genética Brasil, Brasília, DF, Brazil), and cyclophosphamide (CPA, Baxter Hospitalar Ltda., São Paulo, SP, Brazil) were used.

### Protocols *in vivo*

Groups of five mice were weighed before administering the chemicals and treated by gavage (forced feeding) with two combinations of antiretroviral drugs, at three different concentrations each, and two exposure periods (24 h and 48 h).

On the one hand, 3TC did not present any effects administered intraperitoneally to rats at the concentration of 200 mg/kg body weight (BW) assessed by the micronucleus test [33], and therefore we calculated the clinical proportion of the combination TDF + 3TC (2:1) and doubled this concentration, resulting in the lowest concentration of 800 + 400 mg/kg BW. The other concentrations were calculated to be two- and four-fold higher (1600 + 800 and 3200 + 1600 mg/kg BW).

On the other hand, AZT had results at the concentration of 200 mg/kg BW in mice treated intraperitoneally assessed by the micronucleus test [33]. Based on this initial concentration, we calculated the clinical proportion of combivir + EFV (2:1:4), obtaining the lowest concentration of 200 + 100 + 400 mg/kg BW. The other concentrations were calculated to be two- and four-fold higher (400 + 200 + 800 mg/kg BW and 800 + 400 + 1600 mg/kg BW).

The tablets were macerated with a mortar and pestle and diluted in distilled water before administration. Solutions and dilutions were prepared immediately before use. The solvent (distilled water) was used as the negative control, and CPA (50 mg/kg BW) administered intraperitoneally (ip) was used as the positive control.

All the animals were euthanized by cervical dislocation 24 h or 48 h after treatment. The femurs were removed, the proximal epiphysis was cut, and the bone marrow cells from both femurs were flushed with 1 mL fetal calf serum at 37°C. The supernatant was partially discarded and the precipitate, homogenized with a Pasteur pipette, was used for the preparation of slides.

### Comet assay in mice bone marrow

The comet assay was performed using the alkaline method with few modifications [21,31]. Slides previously coated with normal melting point agarose (1.5%) received a homogenate of 15 μL bone marrow cells diluted in 1 mL PBS buffer (pH 7.0) and 130 μL low melting point agarose (0.5%) at 37°C. The material was spread on the slides with coverslips and taken to a cold chamber. After gelation, the coverslips were carefully removed. The slides were immersed in lysis solution protected from light (1% triton X-100, 10% DMSO, 2.5 M NaCl, 100 mM Na_2_EDTA, and 10 mM Tris, pH 10.0) at 4°C for 12–24 h. Subsequently, the slides were incubated with freshly made alkaline solution (300 mM NaOH, 1 mM EDTA, pH > 13) at 4°C for 20 min for DNA unwinding. The slides were kept in cuvettes (protected from light) containing a cold lysis solution (triton X-100, DMSO, and stock lysis solution) for 24 h. Electrophoresis was carried out at 25 V, and the current was adjusted to 300 mA, at 4°C. The slides were exposed to this electrical current in the dark for 30 min. After electrophoresis, the slides were placed in a staining tray, covered with a neutralizing buffer (0.4 M tris-HCl, pH 7.5), and kept in the dark for 5 min. The slides were stained with 20 μL ethidium bromide solution (0.02 mg/mL) and covered with a coverslip. The analysis was performed using a fluorescence microscopy system, Axioplan-Imaging® (Carl Zeiss Light Microscopy, Göttingen, Germany), the Integrated Spectrographic Innovative Software (ISIS) with an excitation filter of 510–560 nm, and a barrier filter of 590 nm, 200x magnification, with 50 nucleoids analyzed per slide, totaling 100 nucleoids per sample.

For the assessment of the genomic damage using the comet assay, the Open Comet^TM^ software, version 1.3 (Cometbio-OpenComet, Singapore) was employed. The nucleoids with completely fragmented heads were not taken into account in our analysis. Of the 17 parameters provided by the software, we selected four, as follows: tail length (TL), percentage of DNA in the tail (%DNA in tail), tail moment (TM), and Olive tail moment (OTM) [34].

The statistical analysis was performed using the software SigmaStat, version 3.5. The mean and standard deviation (SD) of the four aforementioned parameters of each group treated were considered. The analysis of variance (ANOVA) followed by the Tukey’s test *a posteriori* were carried out comparing the treated groups with their respective control groups. The results were considered statistically significant when *p* < 0.05.

### Micronucleus test

The micronucleus test was performed according to Heddle [26]. After sample homogenization, 20-μL aliquots of bone marrow cells prepared as described above were smeared on glass slides, coded for blind analysis, air-dried, and fixed with absolute methanol at room temperature for 5 min. The smears were stained with Giemsa, dibasic sodium phosphate, and monobasic sodium phosphate, and buffered at pH 6.8 for 15 min. After this period, the slides were washed, dried at room temperature, and analyzed in an optical microscope (Olympus BH-2 10x100, Tokyo, Japan), 1000x magnification.

For each animal, two slides were prepared for each concentration of the combinations and 1000 polychromatic erythrocytes (PCE) were counted in each slide, totaling 2000 PCE, to determine the frequency of micronucleated polychromatic erythrocytes (MNPCE) using light microscopy (Olympus BH-2 10 × 100, Tokyo, Japan). Simultaneously, the frequency of normochromatic erythrocytes (NCE) was determined and the PCE/NCE ratio was calculated, allowing inferences about the cytotoxic potential of the drugs tested.

The statistical analysis was carried out using the software SigmaStat, version 3.5. The frequencies of PCE/NCE ratio were compared with the negative control groups using the chi-square test. To compare the cytotoxicity at the two exposure periods, the chi-square test was applied. The frequency of micronuclei per 2000 PCE for each concentration was compared with the negative control group using ANOVA, to infer whether clastogenicity and/or aneugenicity induced by the drugs tested were present. To compare the genotoxicity at the two exposure periods, the Student-t test was performed for each concentration. The results were considered statistically significant when *p* < 0.05.

## Results

All the animals survived the treatments and no clinical signs of toxicity were observed in any treated groups. The frequencies of genomic damage in mice bone marrow and controls are shown in Table 1. Based on the four parameters assessed using the comet assay, neither of the combinations tested significantly induced DNA damage at the exposure periods (24 h and 48 h) compared with their respective negative controls.

**Table 1:** Comet assay analysis in bone marrow cells of mice treated with two antiretroviral combinations and their respective controls.

Treatment	Comet assay parameters^1^ (mean ± SD)			
	TL	% DNA in tail	TM	OTM
**Combivir + EFV**				
**24 h**				
Negative control^2^	9.45 ± 1.98	24.77 ± 5.87	7.72 ± 1.68	4.75 ± 1.01
Positive control^3^	26.44 ± 3.80	74.54 ± 10.77	25.05 ± 3.81	12.96 ± 1.81
200 + 100 + 400 mg/kg	12.25 ± 4.90ª	23.76 ± 5.29ª	9.56 ± 3.11ª	5.97 ± 2.01ª
400 + 200 + 800 mg/kg	12.42 ± 5.74ª	23.42 ± 7.24ª	9.67 ± 4.04ª	6.10 ± 2.45ª
800 + 400 + 1600 mg/kg	10.48 ± 3.23ª	22.19 ± 2.82ª	8.60 ± 2.66ª	5.45 ± 1.67ª
**48 h**				
Negative control	9.45 ± 1.98	24.77 ± 5.87	7.72 ± 1.68	4.75 ± 1.01
Positive control	16.48 ± 3.53	26.09 ± 0.40	10.94 ± 1.44	7.06 ± 0.82
200 + 100 + 400 mg/kg	9.95 ± 3.56ª	18.47 ± 7.85ª	7.16 ± 3.90ª	4.79 ± 2.31ª
400 + 200 + 800 mg/kg	12.05 ± 2.86ª	25.16 ± 3.61ª	9.60 ± 2.61ª	6.15 ± 1.57ª
800 + 400 + 1600 mg/kg	12.26 ± 1.99ª	25.66 ± 7.73ª	9.54 ± 2.44ª	6.00 ± 1.27ª
**TDF + 3TC**				
**24 h**				
Negative control	9.45 ± 1.98	24.77 ± 5.87	7.72 ± 1.68	4.75 ± 1.01
Positive control	26.44 ± 3.80	74.54 ± 10.77	25.05 ± 3.81	12.96 ± 1.81
800 + 400 mg/kg	10.67 ± 3.24ª	23.03 ± 6.86ª	8.30 ± 3.26ª	5.51 ± 1.92ª
1600 + 800 mg/kg	9.79 ± 2.87ª	21.07 ± 8.33ª	7.77 ± 2.97ª	4.77 ± 1.50ª
3200 + 1600 mg/kg	10.15 ± 1.32ª	24.23 ± 4.79ª	7.95 ± 1.20ª	5.05 ± 0.58ª
**48 h**				
Negative control	9.45 ± 1.98	24.77 ± 5.87	7.72 ± 1.68	4.75 ± 1.01
Positive control	16.48 ± 3.53	26.09 ± 0.40	10.94 ± 1.44	7.06 ± 0.82
800 + 400 mg/kg	12.91 ± 3.59ª	30.53 ± 7.46ª	11.21 ± 3.10ª	6.63 ± 1.84ª
1600 + 800 mg/kg	14.06 ± 5.44ª	26.65 ± 13.21ª	11.42 ± 4.92ª	6.98 ± 2.60ª
3200 + 1600 mg/kg	6.74 ± 1.15ª	16.64 ± 2.95ª	5.30 ± 1.08ª	3.38 ± 0.65ª

^1^ TL, tail length; %DNA in tail, percentage of DNA in the tail; TM, tail moment; OTM, Olive tail moment.

^2^ Negative control: distilled water.

^3^ Positive control: cyclophosphamide (50 mg/kg BW).

All the results were compared with their respective control group at the respective exposure period.

^a^ No statistically significant difference compared with the negative control group (*p* > 0.05).

The results obtained for the micronucleus test are presented in Table 2, including the mean MNPCE per 2000 PCE for genotoxicity and the mean PCE/NCE ratio for cytotoxicity. At 24 h, combivir + EFV significantly increased MNPCE frequency, demonstrating its genotoxic potential, and increased the PCE/NCE ratio at all tested concentrations. Compared with the negative control, this combination of drugs showed absence of cytotoxic effect, since cytotoxicity is proven by a decrease in the PCE/NCE ratio. At 48 h of exposure and compared with the negative control, this combination significantly increased MNPCE frequency at the two highest concentrations and increased the PCE/NCE ratio at all concentrations tested, indicating that this combination is non-cytotoxic and exhibited mitogenic potential. Compared with the exposure period of 24 h, at 48 h of exposure, combivir + EFV significantly reduced MNPCE frequency and significantly increased the PCE/NCE ratio at all concentrations tested.

**Table 2:** Frequency of micronucleated polychromatic erythrocytes (MNPCE) and polychromatic and normochromatic erythrocyte (PCE/NCE) ratio in bone marrow of mice treated with two antiretroviral combinations and their respective controls.

Treatment	MNPCE/2000 PCE(mean ±SD)	PCE/NCE(mean ±SD)
**Combivir + EFV**		
**24 h**		
Negative control^1^	3.20 ± 0.84	1.27 ± 0.08
Positive control^2^	27.20 ± 1.30	0.71 ± 0.02
200 + 100 + 400 mg/kg	11.00 ± 1.58^b^	2.17 ± 0.66^b^
400 + 200 + 800 mg/kg	11.60 ± 2.51^b^	1.66 ± 0.16^b^
800 + 400 + 1600 mg/kg	9.80 ± 2.77^b^	1.71 ± 0.19^b^
**48 h**		
Negative control	3.20 ± 0.84	1.27 ± 0.08
Positive control	22.00 ± 1.41	0.78 ± 0.03
200 + 100 + 400 mg/kg	5.60 ± 1.34^a^^,^^d^	3.24 ± 0.63^b^^,^^d^
400 + 200 + 800 mg/kg	6.20 ± 1.48^b^^,^^d^	2.84 ± 0.55^b^^,^^d^
800 + 400 + 1600 mg/kg	6.00 ± 1.58^b^^,^^d^	2.99 ± 0.76^b^^,^^d^
**TDF + 3TC**		
**24 h**		
Negative control	3.20 ± 0.84	1.27 ± 0.08
Positive control	27.20 ± 1.30	0.71 ± 0.02
800 + 400 mg/kg	9.20 ± 1.3^b^	2.83 ± 0.55^b^
1600 + 800 mg/kg	8.00 ± 1.22^b^	2.67 ± 0.15^b^
3200 + 1600 mg/kg	8.60 ± 1.34^b^	3.30 ± 0.34^b^
**48 h**		
Negative control	3.20 ± 0.84	1.27 ± 0.08
Positive control	22.00 ± 1.41	0.78 ± 0.03
800 + 400 mg/kg	4.20 ± 0.45^a^^,^^d^	3.23 ± 0.34^b^^,^^d^
1600 + 800 mg/kg	3.80 ± 0.84^a^^,^^d^	3.14 ± 0.54^b^^,^^d^
3200 + 1600 mg/kg	4.40 ± 0.89^a^^,^^d^	3.30 ± 0.41^b^^,^^c^

^1^ Negative control: distilled water.

^2^ Positive control: cyclophosphamide (50 mg/kg BW).

All the results were compared with their respective control group at the respective exposure period.

^a^ No statistically significant difference compared with the negative control group

(*p* > 0.05).

^b^ Statistically significant difference compared with the negative control group (*p* < 0.05).

^c^ No statistically significant difference compared with the same concentration at 24 h (*p* > 0.05).

^d^ Statistically significant difference compared with the same concentration at 24 h (*p* < 0.05).

At 24 h, TDF + 3TC significantly increased the frequency of MNPCE and the PCE/NCE ratio compared with the negative control, displaying genotoxic effects and non-cytotoxic effects, respectively. At 48 h, the use of this combination of drugs did not lead to significant differences in MNPCE frequency at any tested concentrations compared with the negative control, demonstrating absence of genotoxicity. Additionally, at the same exposure period, this combination caused significant increase in the PCE/NCE ratio at all concentrations tested compared with the negative control, showing no cytotoxicity. Compared with the exposure period of 24 h, at 48 h, this combination significantly reduced MNPCE frequency at all concentrations tested and significantly increased the PCE/NCE ratio at the two lowest concentrations. No changes were observed in the PCE/NCE ratio at the highest concentration tested compared with the exposure period of 24 h.

## Discussion

The aim of this study was to evaluate the cytotoxic and genotoxic effects of two antiretroviral combinations (TDF + 3TC and combivir + EFV) using the comet assay and the micronucleus test.

Neither of the combinations tested exhibited genotoxicity in the comet assay, but both displayed genotoxicity in the micronucleus test at 24 h. In the micronucleus test, DNA damage occurred at chromosomal level, suggesting that drug combinations can induce clastogenic and aneugenic effects. Furthermore, the PCE/NCE ratio was high for both combinations of drugs, suggesting that they can be mitogenic.

EFV, one of the components of the combination combivir + EFV, presents selective cytotoxic effects on various tumor cells, leading to phosphorylation and activation of the p53 tumor suppressor protein. Therefore, it has antitumorigenic and cytostatic potential [35]. This cytotoxic selectivity for tumor cells, or even the interaction of the drugs in the combination combivir + EFV, may have been responsible for the absence of cytotoxic effects observed in this study, inasmuch as our tests were performed in non-tumorous cells. This finding is corroborated by the fact that EFV alone was not cytotoxic to human adipose tissue at low concentrations (0.5 and 4 μM) [36].

In our study, combivir (AZT + 3TC) in combination with EFV displayed mitosis-inducing effects since it increased PCE, thus not exhibiting cytotoxicity. However, the antiretroviral drugs AZT and 3TC administered isolatedly have previously been shown to be cytotoxic to CrFK cells [37]. Therefore, we suggest that the interaction of these drugs may reduce their cytotoxic effects.

In relation to genotoxicity, at the concentration of 400 mg/kg BW, AZT caused genotoxic effects on the peripheral blood lymphocytes and on liver and kidney cells assessed using the comet assay and induced micronuclei in bone marrow cells [34]. Also, AZT was genotoxic to human H9 lymphoblastoid cells in a dose-dependent manner at the concentrations of 0.05, 0.2, 0.4, 0.8, and 1.2 mM. These findings suggest that DNA damage was caused by alkali-labile lesions and not by double-strand breaks, because the genomic damage occurred only at pH 13.0, and not at pH 12.1 or 8.0 [38]. In another study, AZT was genotoxic and 3TC was not genotoxic when administered to neonatal mice. Finally, the combination AZT + 3TC did not alter the responses observed with AZT alone [33].

Increased damage indexes and frequencies were observed in the brain of mice subchronically treated with 10 mg/kg BW EFV. This finding suggests that this drug might induce genotoxicity in the brain [39].

In our study, the combination combivir + EFV had genotoxic effects at chromosomal level (clastogenicity and/or aneugenicity) at both exposure periods. AZT caused chromosomal aberrations in culture cells of patients that received 1200 mg/day [40], therefore, this drug alone may have contributed to the genotoxicity found in our study. Another possibility is that combivir is responsible for the genotoxicity, since AZT and combivir caused a decrease in the percentage of reticulocytes (RETs) and an increase in the percentage of micronucleated RETs and micronucleated normochromatic erythrocytes in neonatal mice [41].

The combination TDF + 3TC did not induce DNA damage assessed using the comet assay, not causing cytotoxicity in either exposure period, but increased MNPCE frequency at 24 h assessed using the micronucleus test. The induction of mitosis in PCE caused by this combination of drugs, observed by the increase in the PCE/NCE ratio, can be due to the action of 3TC. HepG2 cells treated with 3TC, AZT, and abacavir (ABC) showed slight increases in mitochondrial DNA compared with the control group. On the other hand, TDF had no effect on mitochondrial DNA content in this cell line [42]. Given that 3TC interferes with mitochondrial DNA replication, we suggest that this drug could contribute to cell proliferation, inducing mitosis. In a study that assessed the cytotoxicity profile of TDF on various cell types, this antiretroviral drug exhibited the lowest cytotoxic effects on all cell types tested compared to other nucleoside reverse transcriptase inhibitors (NRTIs) [43]. This absence of cytotoxicity was confirmed in our study even in combination with 3TC.

TDF alone showed no genotoxicity assessed by the Ames test, the chromosomal aberration test using Chinese hamster lung (CHL), and the micronucleus assay [44]. However, AZT and 3TC individually tested showed induction of micronuclei in cultured human lymphocytes in binucleated cells using the cytokinesis-block micronucleus (CBMN) assay [13,14]. In the present study, TDF + 3TC exhibited clastogenic and/or aneugenic effects on MNPCE induction.

Comparing both exposure periods, combivir + EFV and TDF + 3TC increased the PCE/NCE ratio respectively at all concentrations tested and at the two lowest concentrations. At 48 h, combivir + EFV and TDF + 3TC caused decrease in the NCE frequency respectively at all concentrations tested and at the two lowest concentrations. In contrast, MNPCE induction significantly decreased at 48 h at all concentrations tested of both combinations. This decrease may be related to the half-life of the compounds, a parameter that is low in relation to the exposure time: 5–9 h for AZT triphosphate and 11–33 h for 3TC triphosphate [45]. For patients with CYP2B6 516 GG, GT, and TT genotypes, EFV half-lives were 23, 27, and 48 h, respectively [46]. After the administration of 300 to 600 mg TDF to human cohorts, the drug concentrations in the serum decreased rapidly with half-lives between 12 h to 15 h [47]. The elimination of micronucleated cells can occur as a consequence of apoptosis [48–50], which justifies the decrease in MNPCE frequency observed at 48 h. A selective elimination of MNPCE in peripheral blood was found [51], and we suggest that the same mechanism may have occurred in the bone marrow.

In the comet assay neither combination induced DNA damage at all tested concentrations at both exposure periods (24 h and 48 h), whereas in the micronucleus test combivir + EFV induced higher MNPCE frequency than TDF + 3TC at all tested concentrations at both exposure periods. In contrast, the PCE/NCE ratio was higher with the combination TDF + 3TC, indicating greater mitogenic potential at both exposure periods.

Genotoxicity may be related to carcinogenesis and both combinations of drugs tested demonstrated that they are capable of inducing clastogenic and/or aneugenic effects. However, further long-term studies are necessary to evaluate the possible side effects of the different combinations of drugs administered in the lifelong antiretroviral therapy of HIV-infected patients.
